# Central Retinal Artery Occlusion in a Patient with Metabolic Syndrome X

**Published:** 2010-01

**Authors:** Sonja Predrag Cekić, Tatjana Petković, Gordana Ljubomir Stanković-Babić, Jovica Mile Mršić

**Affiliations:** 1 Clinic of Ophthalmology, Clinic Centre, Niš, Serbia; 2 Immunological Laboratory, Clinic Centre, Niš, Serbia

**Keywords:** Occlusion, Central Retinal Artery, MetS, Metabolic Syndrome X

## Abstract

**Purpose:**

To report a case of central retinal artery occlusion (CRAO) in a patient with metabolic syndrome X.

**Case Report:**

A 64 year-old-man presented with abrupt, painless, and severe loss of vision in his left eye. Indirect ophthalmoscopy disclosed signs compatible with CRAO and laboratory investigations revealed erythrocyte sedimentation rate of 74 mm/h, C-reactive protein (CRP) level of 21 mg/l, hyperglycemia, hyperuricemia, hypertriglyceridemia and hypercholesterolemia. Fluorescein angiography and immunological studies excluded other systemic disorders. The patient met the full criteria of the National Cholesterol Education Program for metabolic syndrome X.

**Conclusion:**

In addition to different vascular complications such as stroke, and cardiovascular disease, metabolic syndrome X may be associated with retinal vascular occlusions.

## INTRODUCTION

Central retinal artery occlusion (CRAO) is one of the most urgent and dramatic conditions in ophthalmology. It occurs when blockage in the central retinal artery develops within the optic nerve substance, therefore the site of obstruction is not visible on ophthalmoscopy. The hallmark of retinal artery obstruction is abrupt and painless loss of vision. Amaurosis fugax precedes visual loss in about 10% of patients.^1^ Rarely, in cases associated with arterial spasm, a relapsing and remitting course of visual loss may occur prior to CRAO.^1^,^2^

Within minutes to hours after the obstruction, the fundus may appear relatively normal. A cherry-red spot in the macula is typical for this condition and is due to thinness of the retinal nerve fiber layer in the fovea allowing transmission of the normally perfused choroid underneath. The retina becomes opaque elsewhere and blocks the brownish-red color of the choroid.

Conditions associated with CRAO include atherosclerotic cardiovascular disease (CVD); coagulopathic conditions; oncologic, radiologic and medical procedures; systemic vasculitis and infections; local trauma; and local ocular conditions such as prepapillary arterial loops, optic nerve drusen, necrotizing herpetic retinitis, orbital mucuromycosis and toxoplasmosis.^3^

The metabolic syndrome X (MetS) is characterized by abdominal obesity, glucose intolerance, dyslipidemia, high blood pressure and hyperuricemia.^4^ According to the National Cholesterol Education Program (NCEP) criteria, MetS is diagnosed when three or more of the following criteria are present: abdominal type obesity, triglyceride level more than 1.7 mmol/l, high-density lipoprotein (HDL) cholesterol <1.0 mmol/l, blood pressure higher than 130/85 mmHg, and fasting glucose> 6.1 mmol/l.^4^,^5^ Many studies have suggested that arteriosclerosis and insulin resistance share a common inflammatory basis by demonstrating raised C-reactive protein (CRP) levels and a direct harmful effect on vascular walls.^6^–^9^

## CASE REPORT

**Figure 1:**
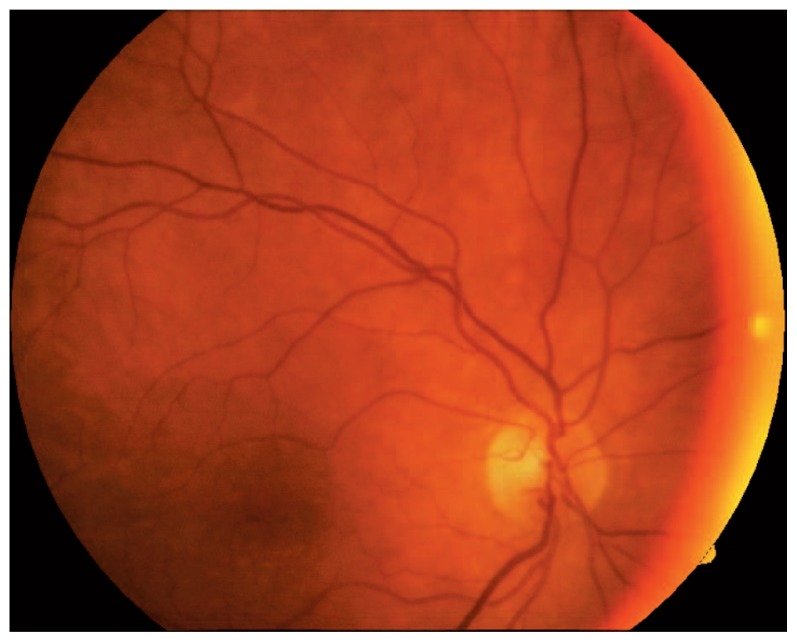
Fundus photograph of the right eye.

**Figure 2:**
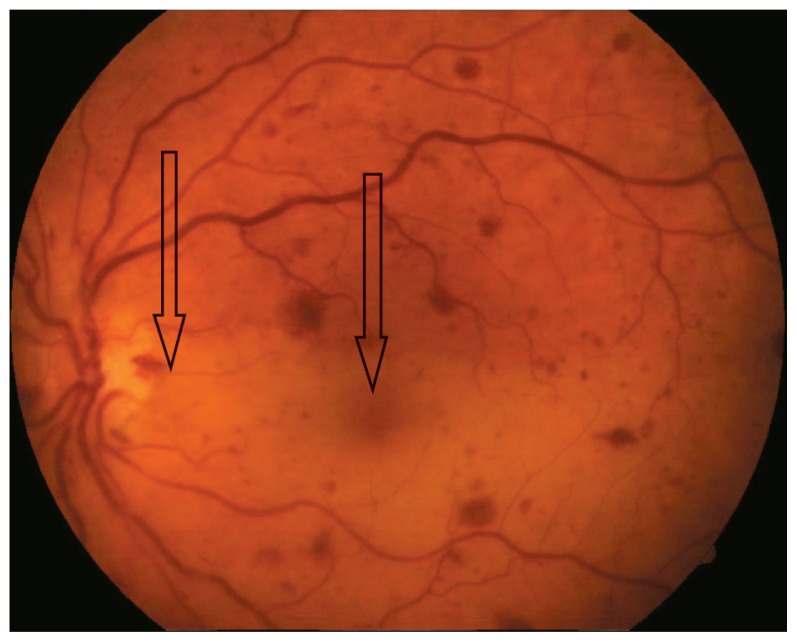
Fundus photograph of the left eye: note the splinter hemorrhages and cherry-red spot.

**Figure 3:**
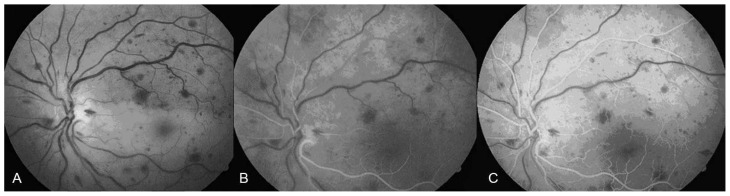
Fluorescein angiography in the left eye of the patient.

A 64 year-old-man presented with abrupt, painless, and severe visual loss to counting fingers at 1 m in his left eye; best-corrected visual acuity was 20/20 in his right eye. He had noted temporary visual loss of a few seconds’ duration in the same eye a few days before total visual loss. Intraocular pressure was 19 mmHg and the anterior segment was normal in both eyes, but afferent pupillary defect was present in the left eye. The right fundus was normal (Fig. 1), however in the left eye, ophthalmoscopy and fluorescein angiography showed narrowing of retinal arteries, boxcarring and fragmentation of blood flow in the retinal arterioles, the retina was opaque and a cherry-red spot was present (Fig. 2). Angiography revealed a prolonged arm-retina time, prolonged filling of retinal arteries, and a long arteriovenous time, as well as late staining of the optic nerve head with peripapillary splinter hemorrhages and disseminated retinal hemorrhages in all quadrants (Fig. 3).

The following laboratory tests were raised above normal limits: fibrinogen 6.1 g/l, CRP 34 mg/l, serum glucose 6.9 mmol/l, HbA1C 7.2, uric acid 451.8 mol/l, total cholesterol 7.5 mmol/l, HDL 0.9 mmol/l, low-density lipoprotein (LDL) 5.0 mmol/l, triglycerides 2.1 mmol/l, and erythrocyte sedimentation rate 42 mm/h. Complete blood count (red blood cells, white blood cells and platelets) was normal. Prothrombine time was 85% and the activated partial thromboplastin time was 26 seconds. Rheumatic factor, antinuclear antibodies, anti-neutrophil cytoplasmic antibodies, immune complexes, and antiphospholipid antibodies were negative.

Clinical examination revealed abdominal obesity and systemic hypertension (160/110 mmHg). A color Doppler scan of the carotid arteries showed bilateral stenosis, 45% on the right side and 64% on the left. Holter monitoring revealed normal cardiac rhythm.

## DISCUSSION

This case report may reflect the need for changing the approach to the patient with CRAO and to point out that complex metabolic changes may be involved in its pathogenesis. Abrupt unilateral visual loss is a symptom in different ophthalmic disorders and is most frequently seen in retrobulbar optic neuritis, anterior ischemic optic neuropathy, severe commotio retinae, necrotizing herpetic retinitis, central or branch retinal vein occlusion (CRVO or BRVO) and CRAO.^2^,^3^ Typical findings in fundus examination and fluorescein angiography help to exclude diagnoses other than CRAO.

The disseminated retinal hemorrhages present in all retinal quadrants in our patient and peripapillary splinter hemorrhages may point toward vasculitis as the etiology for the vaso-occlusive phenomenon. However, standard and immunological laboratory investigations excluded systemic vasculitis, hypercoagulability states and anti-phospholipid antibody syndrome, but suggested a metabolic disorder due to the presence of abdominal obesity, diabetes mellitus, dyslipidemia, high blood pressure and hyperuricemia. The patient reported herein demonstrated all five NCEP criteria for MetS.

The raised proinflammatory marker levels (CRP, fibrinogen) in the MetS are also classic risk factors for CVD and stroke. In the last decade, low grade inflammation has been identified as a pivotal pathogenic factor for development of atherosclerosis and has been shown to predict myocardial infarction and stroke in patients with pre-existing CVD.^7^–^11^ CRP has been investigated extensively in the context of atherosclerosis and vascular complications. Increased CRP is associated with an increased risk of CVD. CRP may also be an important marker for complications of MetS such as CRAO.^12^–^14^ It has been suggested that inflammation might be an important trigger for acute coronary events related to plaque rupture rather than a promoter of chronic atherosclerosis. It has also been proposed that CRP might play an atherogenic role through interaction with low density lipoproteins. CRP level is an important marker in the follow-up of all conditions related to atherosclerosis.^9^–^15^

In summary, the case presented herein demonstrates that CRAO may be associated with MetS and result in profound visual loss. The vascular occlusion is multifactorial and often requires a multidisciplinary approach. It is closely associated with CVD and stroke. Inflammation seems to be an important trigger for CVD, stroke and MetS.
